# Synthesis and crystal structure of *catena*-poly[[bis(pyridine-4-carbonitrile-κ*N*^1^)cobalt(II)]-di-μ-thiocyanato-κ^2^*N*:*S*;κ^2^*S*:*N*]

**DOI:** 10.1107/S2056989026009825

**Published:** 2026-09-25

**Authors:** Christian Näther

**Affiliations:** a Institut für Anorganische Chemie, Universität Kiel, Germany; University of Aberdeen, United Kingdom

**Keywords:** synthesis, cobalt thio­cyanate, pyri­dine-4-car­bo­ni­trile, chain compound, crystal structure

## Abstract

The asymmetric unit of the title compound, [Co(NCS)_2_(C_6_H_4_N_2_)_2_]_*n*_ (C_6_H_4_N_2_ = pyri­dine-4-car­bo­ni­trile), consists of two crystallographically independent cobalt cations that are located on centers of inversion as well as two independent thio­cyanate anions and two independent pyri­dine-4-car­bo­ni­trile coligands that occupy general positions. Each of the cobalt cations is sixfold coordinated by two N- and two S-bonding thio­cyanate anions and two pyri­dine-4-car­bo­ni­trile coligands in an slightly distorted CoN_4_S_2_ octa­hedral geometry with an all-*trans* coordination. The cobalt cations are linked by pairs of μ-1,3-bridging anionic ligands into linear chains. Powder X-ray diffraction (PXRD) experiments indicate that the title compound was obtained as a pure phase. Moreover, comparison of the experimental X-ray powder pattern of the residue obtained after water removal from [Co(NCS)_2_(C_6_H_4_N_2_)_2_(H_2_O)_2_] reported recently [Näther (2026 ▸).*Acta Cryst.* E**82**, 441–445] with the calculated powder pattern of the title compound proves that the latter crystalline phase has formed and indicates that a second modification with the same composition as that of the title compound might exist.

## Abstract 2

In the crystal structure of the title compound, the cobalt cations are octa­hedrally coordinated by two N- and two S-bonding thio­cyanate anions and two pyri­dine-4-car­bo­ni­trile ligands and are linked by pairs of μ-1,3-bridging anionic ligands into infinite linear chains. No directional inter­molecular inter­actions are observed between the chains.

## 1. Chemical context

For many years, we and others have been inter­ested in the synthesis, crystal structures and magnetic properties of transition-metal thio­cyanate and seleno­cyanate coordination polymers in which the metal cations are linked by μ-1,3-bridging anionic ligands into one-dimensional or two-dimensional networks (Näther & Greve, 2003 ▸; Wöhlert *et al.*, 2013 ▸; Neumann *et al.*, 2018 ▸). In this context compounds based on Co^II^ are of special inter­est, because they can show one-dimensional or three-dimensional ferromagnetic ordering (Lescouëzec *et al.*, 2005 ▸; Mautner *et al.*, 2018 ▸; Wöhlert *et al.*, 2012 ▸; 2013 ▸; Rams *et al.*, 2020 ▸). In some cases such compounds are difficult to prepare and we therefore developed an alternative strategy in which precursor compounds with only terminally N-bonding thio­cyanate anions and weakly bound coligands are heated. This frequently leads to the formation of compounds in which the metal cations are linked by bridging anionic ligands (Näther & Greve, 2003 ▸; Wriedt & Näther, 2010 ▸).

In this context, we have recently reported on a discrete aqua complex with the composition Co(NCS)_2_(C_6_H_4_N_2_)_2_(H_2_O)_2_ (C_6_H_4_N_2_ = pyri­dine-4-car­bo­ni­trile) in which the metal cations are octa­hedrally coordinated by two N-bonding thio­cyanate anions, two pyri­dine-4-car­bo­ni­trile ligands and two water mol­ecules. Thermogravimetric measurements reveal, that this complex decomposes in separate steps, in which compounds with the composition Co(NCS)_2_(C_6_H_4_N_2_)_2_ and Co(NCS)_2_(C_6_H_4_N_2_) are formed as inter­mediates. PXRD measurements of these residues reveal that crystalline phases have formed and that the residue obtained after the first mass loss consists of a mixture of two different crystalline phases, of which one might be isotypic to the corresponding Mn compound Mn(NCS)_2_(C_6_H_4_N_2_)_2_ (Wellm *et al.*, 2020 ▸).
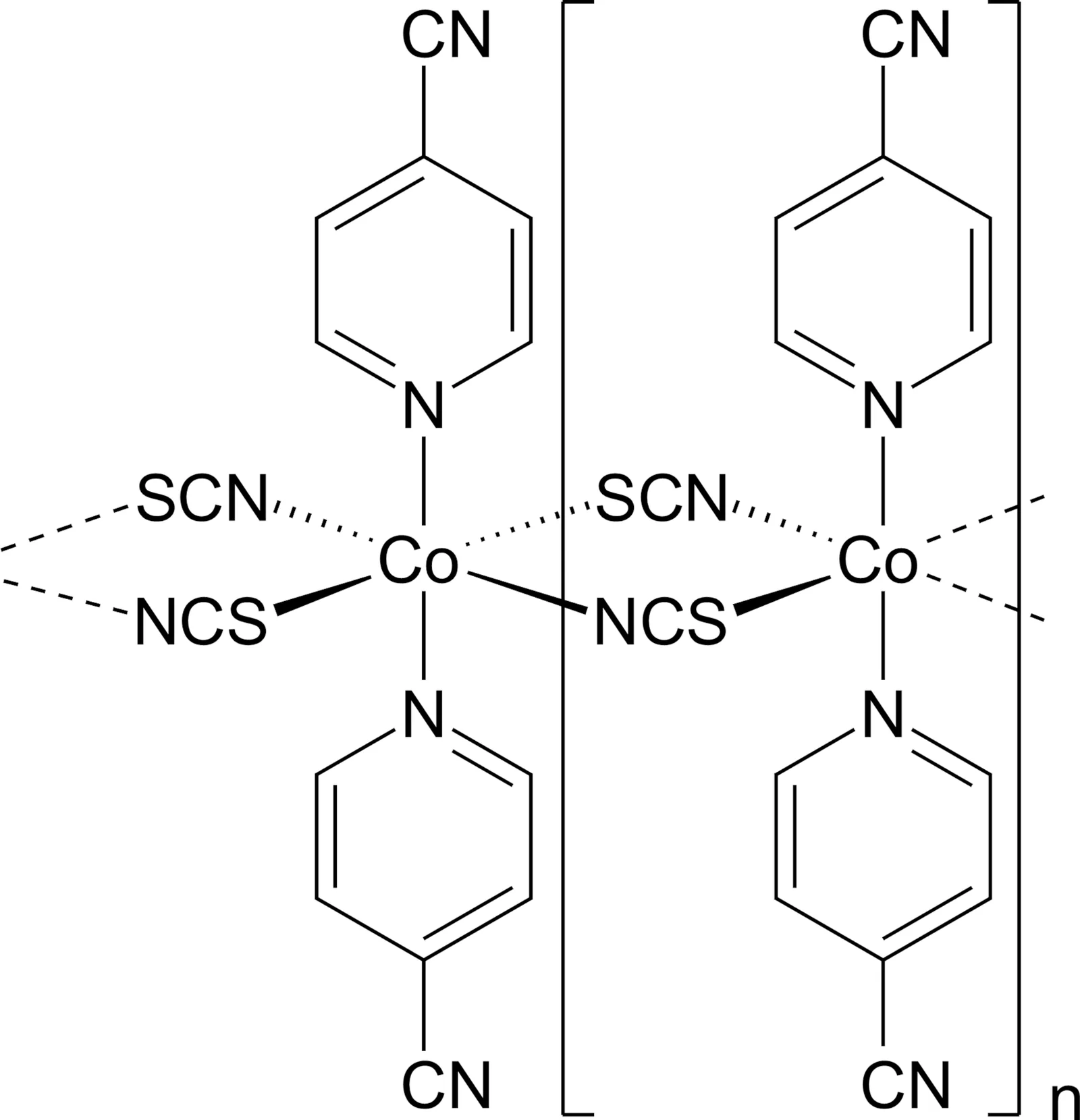


Starting from these results we tried to prepare single crystals of the two pyri­dine-4-car­bo­ni­trile-deficient coordination compounds, which led to the formation of crystals of the title compound, Co(NCS)_2_(C_6_H_4_N_2_)_2_ (**I**), which were characterized by single crystal X-ray diffraction.

## 2. Structural commentary

The asymmetric unit of (**I**) consists of two crystallographically independent cobalt cations that occupy centers of inversion as well as two crystallographically independent thio­cyanate anions and two crystallographically independent pyri­dine-4-car­bo­ni­trile coligands that are located in general positions in space group *P*2_1_/*c*. Each cobalt cation is sixfold coordinated by pairs of N- and S-bonding thio­cyanate anions and two pyri­dine-4-car­bo­ni­trile coligands that coordinate with the pyridine atom to the metal centers in an all-*trans* CoN_4_S_2_ octa­hedral coordination environment (Fig. 1 ▸). Bond lengths and angles are comparable for both cobalt cations and deviate only slightly from the ideal values, which shows that the octa­hedra are slightly distorted (Table 1 ▸). The cobalt cations are linked by pairs of μ-1,3-bridging anionic ligands into linear chains that propagate along the [101] direction (Fig. 2 ▸).

It is noted that the title compound is not isotypic to the corresponding Mn compound already reported in the literature, which crystallizes in the monoclinic space group *P*2_1_/*n* with *a* = 5.7311 (4) Å, *b* =17.86665 (15) Å, *c* = 8.5822 (5) Å and β = 107.624° [*V* = 837.5 (3) Å^3^ at 195 K] (Wellm *et al.*, 2020 ▸). Surprisingly, a similar unit cell with the same space group can also be found for the title compound if some of the weak reflections are omitted. However, if the structure is refined in this smaller unit cell, the six-membered rings of the pyri­dine-4-car­bo­ni­trile are disordered over two orientations, which clearly shows that the larger unit cell is correct. In contrast, in the Mn compound with half of the unit-cell volume no disorder is observed, which shows that this unit cell is correct and that this modification is different from that of the title compound.

## 3. Supra­molecular features

In the extended structure of (**I**), the [101] chains are packed in a way that is reminiscent of a sandwich-herringbone arrangement (Fig. 3 ▸). There are a number of C—H⋯N and C—H⋯S contacts (Table 2 ▸) but with long H⋯N and H⋯S distances and with angles far from linearity, indicating that these are very weak inter­actions.

## 4. Database survey

It is noted that several coordination compounds with pyri­dine-4-car­bo­ni­trile and transition metal thio­cyanates are already reported in the CSD [version 5.43, last update January 2026 (Groom *et al.*, 2016 ▸) searched using CONQUEST (Bruno *et al.*, 2002 ▸)]. Most of them are combined with Mn(NCS)_2_. This includes discrete complexes with the composition Mn(NCS)_2_(C_6_H_4_N_2_)_4_ (CSD refcode: OJEDOZ; Wellm *et al.*, 2020 ▸) and Mn(NCS)_2_(C_6_H_4_N_2_)_2_(H_2_O)_2_ (OJEFAN and OJEFAN01; Wellm *et al.*, 2020 ▸), which crystallizes in two different modifications. Discrete aqua complexes with the composition Mn(NCS)_2_(C_6_H_4_N_2_)_2_(H_2_O)_2_ and additional pyri­dine-4-car­bo­ni­trile as solvate mol­ecules are also known (OJEFER and OJEFUH; Wellm *et al.*, 2020 ▸). Compounds with bridging anionic ligands with the composition Mn(NCS)_2_(C_6_H_4_N_2_)_2_ (OJEFIV; Wellm *et al.*, 2020 ▸) and two isomers of Mn(NCS)_2_(C_6_H_4_N_2_) (OJEDUF and OJEDUF01; Wellm *et al.*, 2020 ▸) are also known.

With nickel, two compounds built up of discrete complexes are reported, *viz*. Ni(NCS)_2_(C_6_H_4_N_2_)_4_ (UBUBOL; Clegg & Harrington, 2016 ▸) and Ni(NCS)_2_(C_6_H_4_N_2_)_2_(H_2_O)_2_ (Näther, 2026 ▸). Fe(NCS)_2_(C_6_H_4_N_2_)_2_(H_2_O)_2_·(C_6_H_4_N_2_)_2_ (KAPQEA; Jochim *et al.*, 2017 ▸) also consists of discrete complexes with additional pyri­dine-4-car­bo­ni­trile mol­ecules located in cavities of this structure.

Moreover, compounds with the composition Cd(NCS)_2_(C_6_H_4_N_2_)_2_ (WUCLUB; Chen *et al.*, 2002 ▸) and Cu(NCS)_2_(C_6_H_4_N_2_)_2_ (ABOVOF; Handy *et al.*, 2017 ▸) are known which exhibit the same ratio between metal thio­cyanate and pyri­dine-4-car­bo­ni­trile as observed in the title compound. In both compounds the metal cations are linked by the anionic ligands into chains, but none of these compounds is isotypic to the title compound. The cadmium compound is isotypic to the corresponding manganese compound mentioned above (OJEFIV; Wellm *et al.*, 2020 ▸). Finally, in Cd(NCS)_2_(C_6_H_4_N_2_) (WUCMAI; Chen *et al.*, 2002 ▸) the pyri­dine-4-car­bo­ni­trile ligand acts as a bridging ligand. This compound is isotypic to one of the two modifications of Mn(NCS)_2_(C_6_H_4_N_2_) (OJEDUF01; Wellm *et al.*, 2020 ▸).

## 5. Synthesis and crystallization

Co(NCS)_2_ and pyri­dine-4-car­bo­ni­trile were purchased from Sigma-Aldrich. 1.00 mmol (175.2 mg) Co(NCS)_2_ and 2.00 mmol (208.2 mg) pyri­dine-4-car­bo­ni­trile were stirred in 1 ml of ethanol for 1 d. The precipitate was filtered off and dried in air. Single crystals of (**I**) in the form of light-red blocks were obtained using the same amount of reactants without stirring.

The asymmetric CN stretching vibration of the thio­cyanate anions is observed at 2102 cm^−1^ in the IR spectrum, in agreement with the bridging coordination of the anionic ligands (Fig. 4 ▸). Powder X-ray diffraction (PXRD) experiments show that the title compound was obtained as a pure phase (Fig. 5 ▸). Moreover, comparison of the experimental X-ray powder pattern of the residue obtained after water removal from Co(NCS)_2_(C_6_H_4_N_2_)_2_(H_2_O)_2_ reported recently (Näther, 2026 ▸) with the calculated powder pattern of the title compound indicate that the title compound has formed in a mixture with a second and unknown modification of Co(NCS)_2_(C_6_H_4_N_2_)_2_ (Fig. 6 ▸).

Powder X-ray diffraction measurements were performed using a Stoe STADI P transmission powder diffractometer with Cu *K*α_1_ radiation (λ = 1.540598 Å), a Johann-type Ge(111) monochromator and a MYTHEN 1K detector from Dectris.

IR spectroscopy was done using an ATI Mattson Genesis Series FTIR Spectrometer, control software: *WINFIRST*, from ATI Mattson in ATR mode.

## 6. Refinement

Crystal data, data collection and structure refinement details are summarized in Table 3 ▸. The C-bound hydrogen atoms were positioned with idealized geometry and were refined with *U*_iso_(H) = 1.2*U*_eq_(C) using a riding model.

## Supplementary Material

Crystal structure: contains datablock(s) I. DOI: 10.1107/S2056989026009825/hb8260sup1.cif

Structure factors: contains datablock(s) I. DOI: 10.1107/S2056989026009825/hb8260Isup2.hkl

CCDC reference: 2589650

Additional supporting information:  crystallographic information; 3D view; checkCIF report

## Figures and Tables

**Figure 1:**
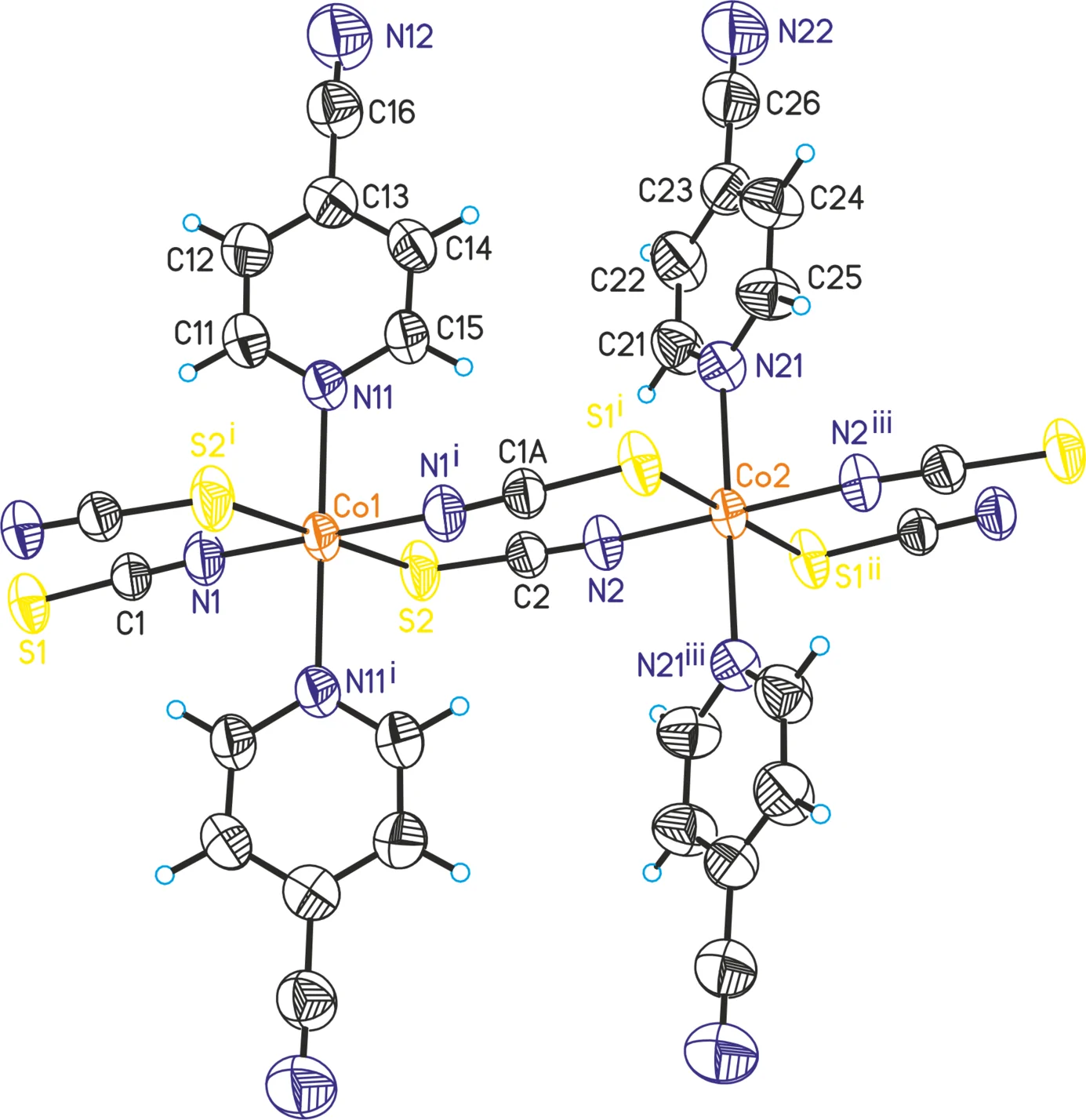
Crystal structure of (**I**) with labeling and displacement ellipsoids drawn at the 50% probability level. Symmetry codes: (i) *x* − 1, *y*, *z -* 1; (ii) −*x* + 2, −*y* + 1, −*z* + 1; (iii) −*x* + 1, −*y* + 1, −*z*.

**Figure 2:**
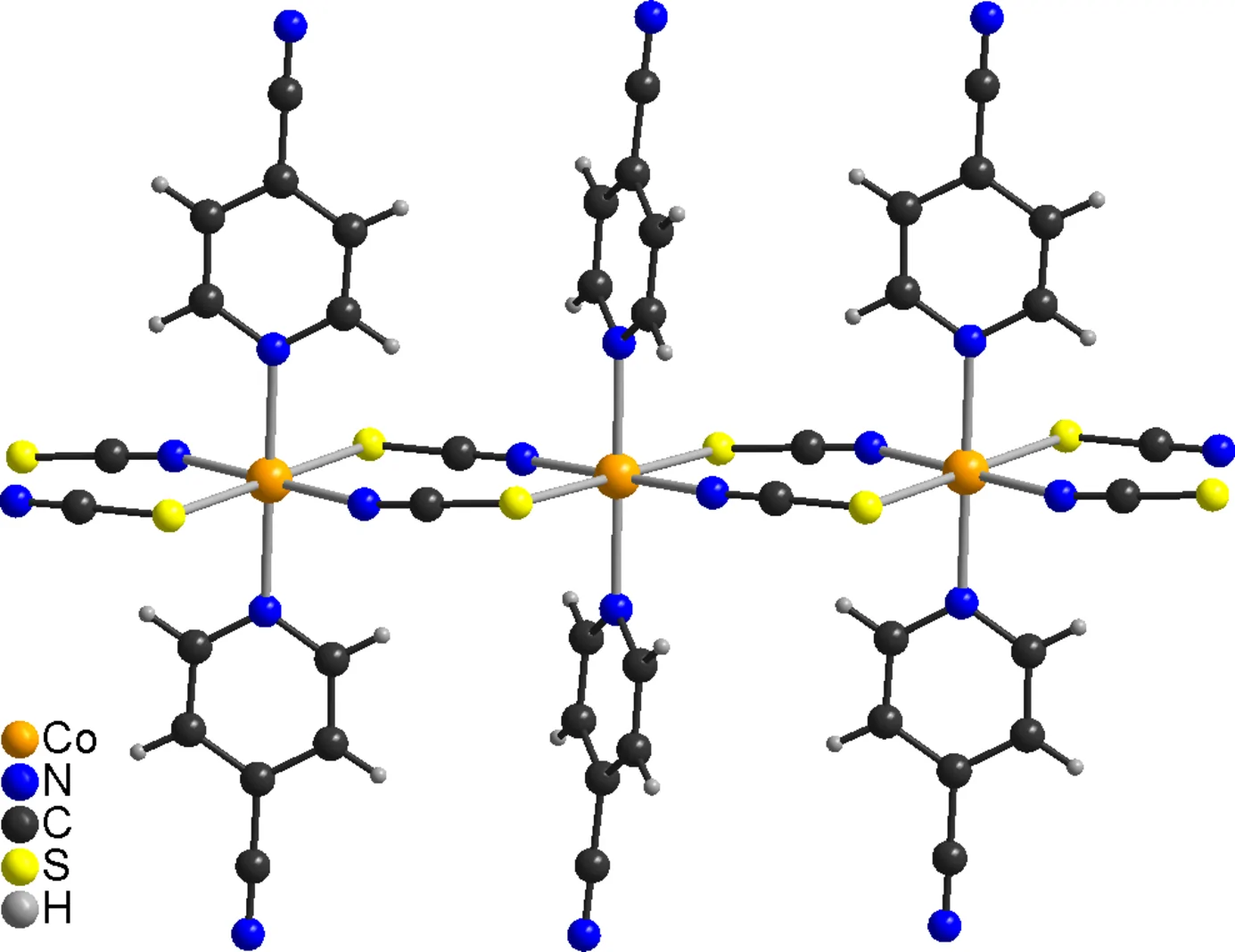
Fragment of the crystal structure of (**I**) with a view of part of a chain.

**Figure 3:**
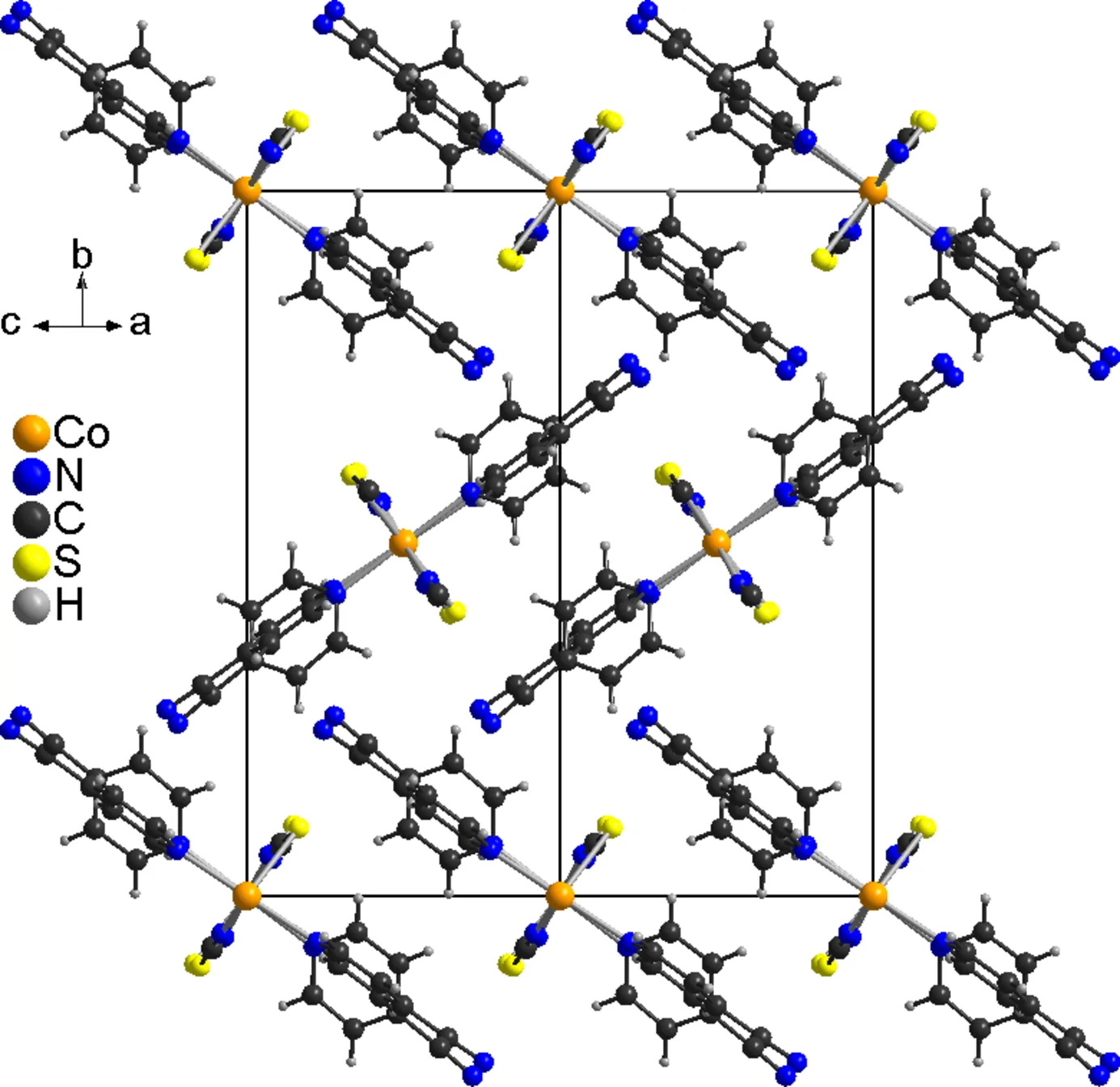
Crystal structure of (**I**) in a view along the [101] direction.

**Figure 4:**
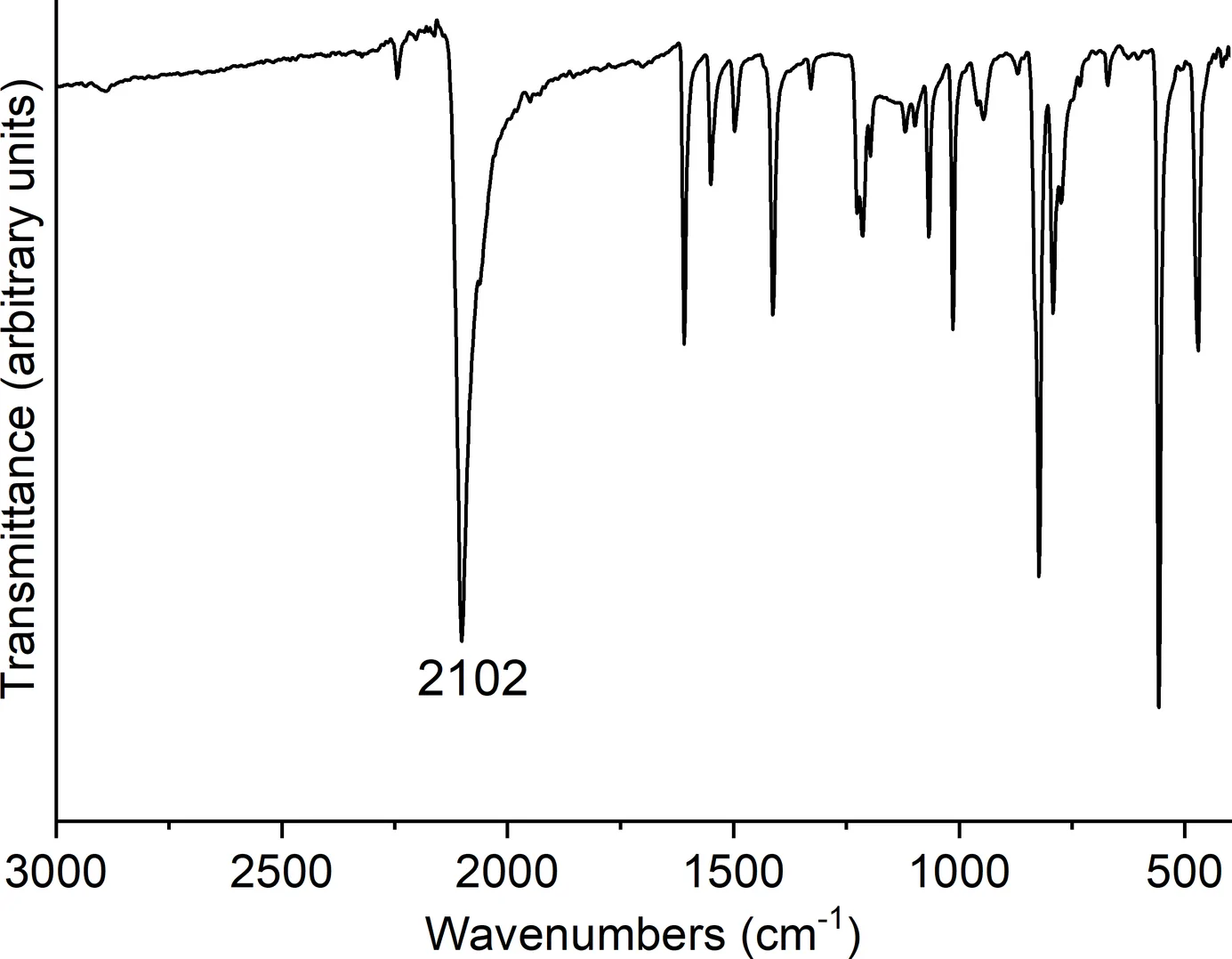
IR spectrum of (**I**) with the CN stretching vibration of the thio­cyanate anion shown.

**Figure 5:**
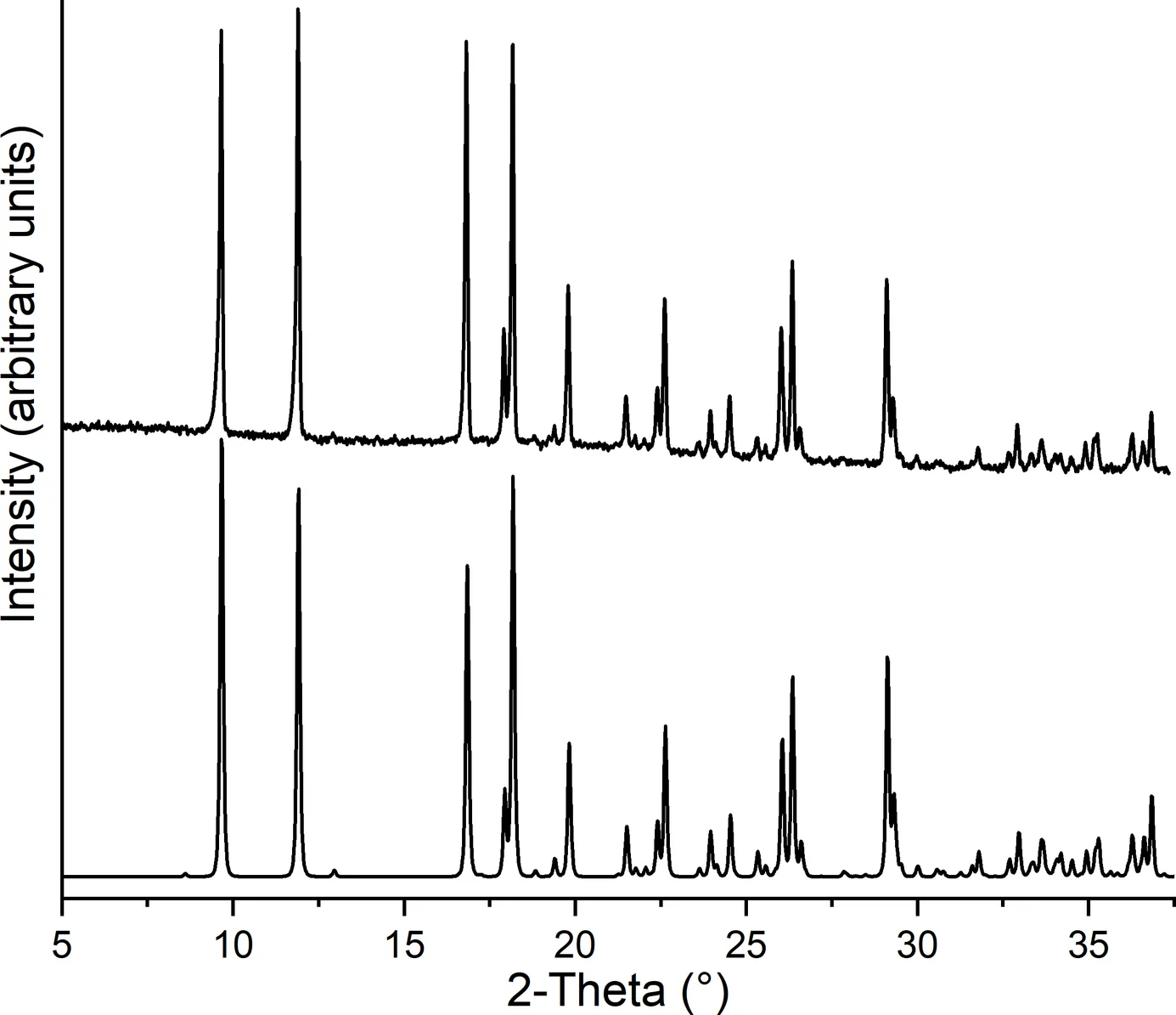
Experimental (top) and calculated (bottom) PXRD patterns for (**I**).

**Figure 6:**
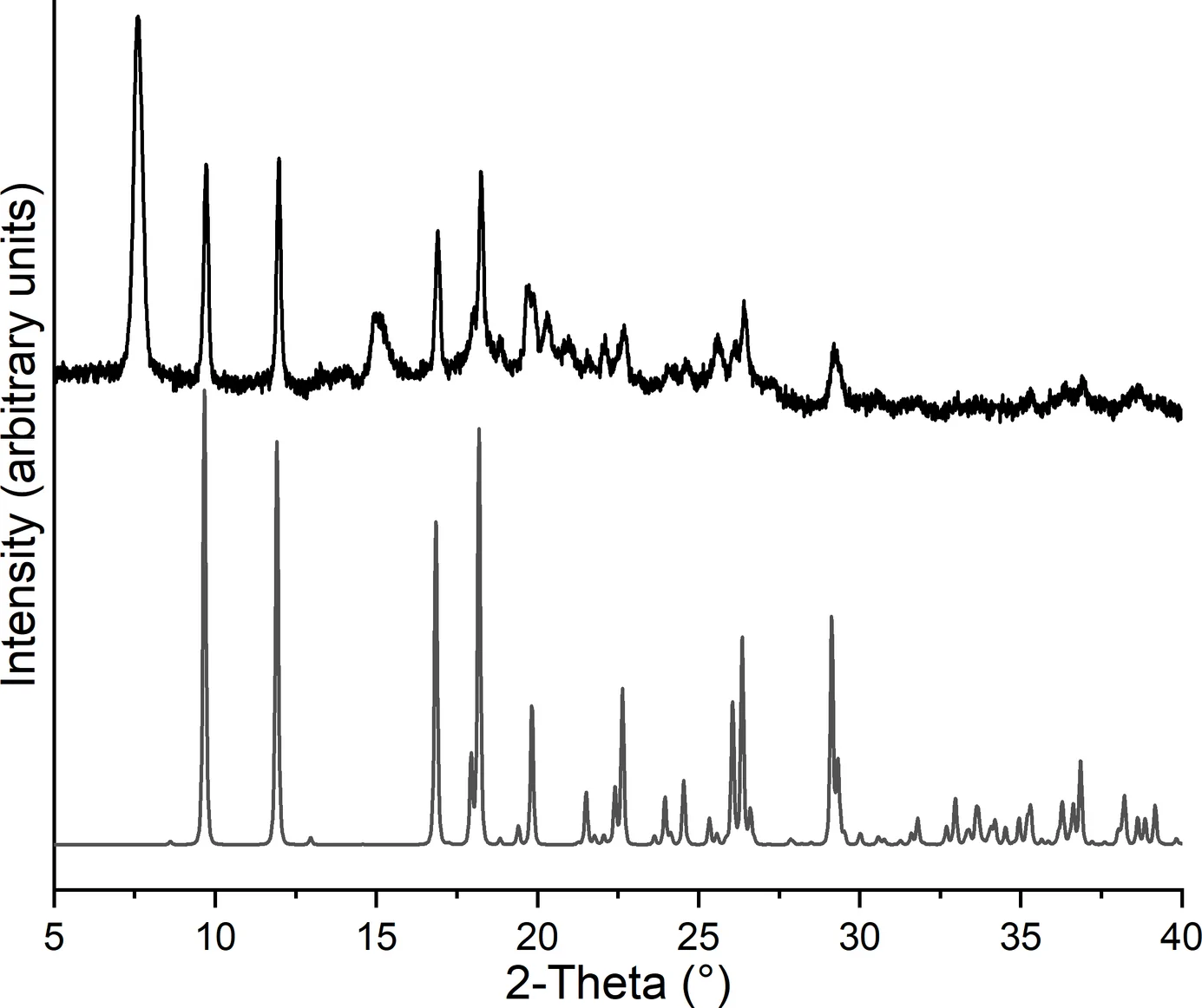
Experimental (top) PXRD pattern of Co(NCS)_2_(C_6_H_4_N_2_)_2_ obtained by water removal from Co(NCS)_2_(C_6_H_4_N_2_)_2_(H_2_O)_2_ (Wellm *et al.*, 2020 ▸) and calculated (bottom) powder pattern for (**I**).

**Table 1:** Selected geometric parameters (Å, °)

Co1—N1	2.068 (2)	Co2—S1^i^	2.6178 (6)
Co1—S2	2.5836 (7)	Co2—N2	2.0462 (19)
Co1—N11	2.181 (2)	Co2—N21	2.185 (2)
			
N1—Co1—S2	86.30 (6)	N2—Co2—S1^ii^	92.99 (6)
N1^ii^—Co1—S2	93.70 (6)	N2—Co2—S1^i^	87.01 (6)
N1^ii^—Co1—N11	89.37 (8)	N2—Co2—N21^iii^	89.60 (8)
N1—Co1—N11	90.63 (8)	N2—Co2—N21	90.40 (8)
N11^ii^—Co1—S2	92.18 (6)	N21—Co2—S1^i^	88.87 (6)
N11—Co1—S2	87.81 (6)	N21—Co2—S1^ii^	91.13 (6)

Symmetry codes: (i) 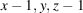; (ii) 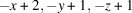; (iii) 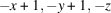.

**Table 2:** Hydrogen-bond geometry (Å, °)

*D*—H⋯*A*	*D*—H	H⋯*A*	*D*⋯*A*	*D*—H⋯*A*
C11—H11⋯N1	0.93	2.63	3.126 (4)	114
C15—H15⋯N1^ii^	0.93	2.59	3.106 (3)	115
C21—H21⋯N2	0.93	2.61	3.134 (3)	116
C22—H22⋯N12^iv^	0.93	2.67	3.287 (4)	124
C24—H24⋯S2^v^	0.93	2.98	3.813 (3)	150
C25—H25⋯N2^iii^	0.93	2.55	3.065 (4)	115

Symmetry codes: (ii) 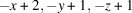; (iii) 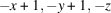; (iv) 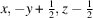; (v) 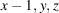.

**Table 3:** Experimental details

Crystal data	
Chemical formula	[Co(NCS)_2_(C_6_H_4_N_2_)_2_]
*M*_r_	383.31
Crystal system, space group	Monoclinic, *P*2_1_/*c*
Temperature (K)	300
*a*, *b*, *c* (Å)	11.1023 (4), 18.2814 (8), 8.9315 (5)
β (°)	112.436 (3)
*V* (Å^3^)	1675.57 (14)
*Z*	4
Radiation type	Mo *K*α
μ (mm^−1^)	1.28
Crystal size (mm)	0.17 × 0.12 × 0.09

Data collection	
Diffractometer	Stoe IPDS 2
Absorption correction	Numerical (*X-RED* and *X-SHAPE*; Stoe, 2008 ▸)
*T*_min_, *T*_max_	0.710, 0.845
No. of measured, independent and observed [*I* > 4u(*I*)] reflections	16251, 3288, 2401
*R*_int_	0.043
(sin θ/λ)_max_ (Å^−1^)	0.617

Refinement	
*R*[*F*^2^ > 2σ(*F*^2^)], *wR*(*F*^2^), *S*	0.036, 0.088, 1.07
No. of reflections	3288
No. of parameters	211
H-atom treatment	H-atom parameters constrained
Δρ_max_, Δρ_min_ (e Å^−3^)	0.38, −0.25

Computer programs: *X-AREA* and *X-RED32* (Stoe, 2008 ▸), *SHELXT* (Sheldrick, 2015*a* ▸), *SHELXL2016/6* (Sheldrick, 2015*b* ▸), *OLEX2* (Dolomanov *et al.*, 2009 ▸), *SHELXTL* (Sheldrick, 2008 ▸), *DIAMOND* (Brandenburg, 1999 ▸) and *publCIF* (Westrip, 2010 ▸).

## supplementary crystallographic information

### catena-Poly[[bis(pyridine-4-carbonitrile-κN1)cobalt(II)]-di-µ-thiocyanato-κ2N:S;κ2S:N] Crystal data

**Table:**

[Co(NCS)_2_(C_6_H_4_N_2_)_2_]	*F*(000) = 772
*M**_r_* = 383.31	*D*_x_ = 1.520 Mg m^−^^3^
Monoclinic, *P*2_1_/*c*	Mo *K*α radiation, λ = 0.71073 Å
*a* = 11.1023 (4) Å	Cell parameters from 13003 reflections
*b* = 18.2814 (8) Å	θ = 2.0–28.4°
*c* = 8.9315 (5) Å	µ = 1.28 mm^−^^1^
β = 112.436 (3)°	*T* = 300 K
*V* = 1675.57 (14) Å^3^	Block, light red
*Z* = 4	0.17 × 0.12 × 0.09 mm

### catena-Poly[[bis(pyridine-4-carbonitrile-κN1)cobalt(II)]-di-µ-thiocyanato-κ2N:S;κ2S:N] Data collection

**Table:**

Stoe IPDS 2 diffractometer	3288 independent reflections
Radiation source: sealed X-ray tube, 12 x 0.4 mm long-fine focus	2401 reflections with *I* > 4u(*I*)
Plane graphite monochromator	*R*_int_ = 0.043
Detector resolution: 6.67 pixels mm^-1^	θ_max_ = 26.0°, θ_min_ = 2.0°
ω scans	*h* = −13→13
Absorption correction: numerical (X-Red and X-Shape; Stoe, 2008)	*k* = −21→22
*T*_min_ = 0.710, *T*_max_ = 0.845	*l* = −11→11
16251 measured reflections	

### catena-Poly[[bis(pyridine-4-carbonitrile-κN1)cobalt(II)]-di-µ-thiocyanato-κ2N:S;κ2S:N] Refinement

**Table:**

Refinement on *F*^2^	0 restraints
Least-squares matrix: full	Hydrogen site location: inferred from neighbouring sites
*R*[*F*^2^ > 2σ(*F*^2^)] = 0.036	H-atom parameters constrained
*wR*(*F*^2^) = 0.088	*w* = 1/[σ^2^(*F*_o_^2^) + (0.0406*P*)^2^ + 0.2371*P*] where *P* = (*F*_o_^2^ + 2*F*_c_^2^)/3
*S* = 1.07	(Δ/σ)_max_ < 0.001
3288 reflections	Δρ_max_ = 0.38 e Å^−^^3^
211 parameters	Δρ_min_ = −0.25 e Å^−^^3^

### catena-Poly[[bis(pyridine-4-carbonitrile-κN1)cobalt(II)]-di-µ-thiocyanato-κ2N:S;κ2S:N] Special details

**Table:**

Geometry. All esds (except the esd in the dihedral angle between two l.s. planes) are estimated using the full covariance matrix. The cell esds are taken into account individually in the estimation of esds in distances, angles and torsion angles; correlations between esds in cell parameters are only used when they are defined by crystal symmetry. An approximate (isotropic) treatment of cell esds is used for estimating esds involving l.s. planes.

### catena-Poly[[bis(pyridine-4-carbonitrile-κN1)cobalt(II)]-di-µ-thiocyanato-κ2N:S;κ2S:N] Fractional atomic coordinates and isotropic or equivalent isotropic displacement parameters (Å^2^)

**Table:**

	*x*	*y*	*z*	*U*_iso_*/*U*_eq_	
Co1	1.000000	0.500000	0.500000	0.04672 (14)	
Co2	0.500000	0.500000	0.000000	0.04134 (13)	
N1	1.17221 (19)	0.44202 (12)	0.6006 (2)	0.0515 (5)	
C1	1.2795 (2)	0.42552 (13)	0.6692 (3)	0.0416 (5)	
S1	1.43258 (5)	0.40342 (4)	0.76597 (8)	0.05299 (18)	
N2	0.67531 (18)	0.44698 (11)	0.0948 (2)	0.0480 (5)	
C2	0.7798 (2)	0.42780 (13)	0.1701 (3)	0.0429 (5)	
S2	0.92796 (6)	0.40021 (4)	0.27846 (8)	0.0588 (2)	
N11	0.91226 (18)	0.43418 (12)	0.6354 (2)	0.0515 (5)	
C11	0.9848 (3)	0.40288 (19)	0.7757 (4)	0.0703 (8)	
H11	1.074005	0.411988	0.818576	0.084*	
C12	0.9339 (3)	0.35758 (19)	0.8603 (4)	0.0702 (8)	
H12	0.987944	0.336118	0.957045	0.084*	
C13	0.8022 (3)	0.34469 (14)	0.7996 (3)	0.0543 (6)	
C14	0.7265 (2)	0.37688 (15)	0.6556 (3)	0.0563 (6)	
H14	0.636912	0.369148	0.611322	0.068*	
C15	0.7847 (2)	0.42066 (16)	0.5777 (3)	0.0548 (6)	
H15	0.732429	0.441945	0.479851	0.066*	
C16	0.7433 (3)	0.29886 (17)	0.8840 (4)	0.0660 (7)	
N12	0.6942 (3)	0.26440 (17)	0.9497 (4)	0.0947 (9)	
N21	0.42045 (19)	0.42792 (10)	0.1347 (3)	0.0486 (5)	
C21	0.4642 (3)	0.35993 (15)	0.1750 (4)	0.0633 (7)	
H21	0.534751	0.344242	0.151511	0.076*	
C22	0.4097 (3)	0.31194 (17)	0.2500 (4)	0.0708 (8)	
H22	0.443326	0.264979	0.276604	0.085*	
C23	0.3053 (3)	0.33407 (15)	0.2850 (3)	0.0593 (7)	
C24	0.2607 (3)	0.40443 (17)	0.2465 (4)	0.0672 (8)	
H24	0.191268	0.421650	0.270553	0.081*	
C25	0.3206 (3)	0.44875 (15)	0.1719 (4)	0.0624 (7)	
H25	0.289582	0.496223	0.145920	0.075*	
C26	0.2421 (3)	0.28496 (19)	0.3578 (4)	0.0767 (9)	
N22	0.1929 (3)	0.2449 (2)	0.4133 (4)	0.1074 (11)	

### catena-Poly[[bis(pyridine-4-carbonitrile-κN1)cobalt(II)]-di-µ-thiocyanato-κ2N:S;κ2S:N] Atomic displacement parameters (Å^2^)

**Table:**

	*U*^11^	*U*^22^	*U*^33^	*U*^12^	*U*^13^	*U*^23^
Co1	0.0261 (2)	0.0694 (3)	0.0399 (3)	0.0037 (2)	0.00720 (19)	−0.0001 (2)
Co2	0.0275 (2)	0.0449 (2)	0.0449 (3)	0.00325 (17)	0.00625 (18)	0.0008 (2)
N1	0.0356 (11)	0.0663 (13)	0.0450 (12)	0.0026 (10)	0.0068 (9)	−0.0043 (10)
C1	0.0342 (12)	0.0482 (13)	0.0390 (12)	0.0004 (9)	0.0102 (10)	−0.0044 (10)
S1	0.0308 (3)	0.0583 (4)	0.0597 (4)	0.0074 (3)	0.0059 (3)	−0.0112 (3)
N2	0.0322 (10)	0.0524 (11)	0.0503 (12)	0.0033 (9)	0.0058 (9)	−0.0041 (10)
C2	0.0347 (12)	0.0513 (13)	0.0409 (13)	−0.0002 (10)	0.0123 (10)	−0.0039 (11)
S2	0.0333 (3)	0.0803 (5)	0.0525 (4)	0.0156 (3)	0.0050 (3)	−0.0081 (3)
N11	0.0355 (10)	0.0697 (14)	0.0470 (12)	0.0025 (9)	0.0132 (9)	−0.0003 (10)
C11	0.0405 (14)	0.103 (2)	0.0613 (17)	0.0044 (15)	0.0129 (12)	0.0164 (17)
C12	0.0526 (16)	0.092 (2)	0.0625 (18)	0.0084 (15)	0.0186 (14)	0.0204 (16)
C13	0.0560 (16)	0.0497 (14)	0.0602 (16)	−0.0005 (12)	0.0255 (13)	−0.0054 (12)
C14	0.0417 (13)	0.0624 (15)	0.0628 (17)	−0.0056 (12)	0.0177 (12)	−0.0051 (14)
C15	0.0381 (12)	0.0686 (16)	0.0517 (15)	0.0015 (12)	0.0105 (11)	0.0002 (13)
C16	0.0625 (17)	0.0579 (16)	0.075 (2)	−0.0046 (14)	0.0229 (15)	0.0040 (15)
N12	0.085 (2)	0.087 (2)	0.106 (2)	−0.0131 (17)	0.0302 (18)	0.0300 (19)
N21	0.0444 (11)	0.0464 (11)	0.0539 (12)	−0.0002 (9)	0.0174 (10)	−0.0003 (9)
C21	0.0505 (15)	0.0551 (15)	0.086 (2)	0.0088 (12)	0.0282 (14)	0.0146 (15)
C22	0.0577 (16)	0.0540 (16)	0.097 (2)	0.0047 (13)	0.0255 (16)	0.0192 (16)
C23	0.0545 (16)	0.0630 (16)	0.0567 (17)	−0.0097 (13)	0.0171 (13)	0.0045 (14)
C24	0.0701 (19)	0.0651 (17)	0.080 (2)	0.0045 (15)	0.0438 (17)	0.0037 (15)
C25	0.0720 (18)	0.0513 (14)	0.0772 (19)	0.0079 (13)	0.0433 (16)	0.0034 (14)
C26	0.0657 (19)	0.081 (2)	0.081 (2)	−0.0086 (16)	0.0247 (17)	0.0157 (18)
N22	0.087 (2)	0.118 (3)	0.117 (3)	−0.019 (2)	0.038 (2)	0.039 (2)

### catena-Poly[[bis(pyridine-4-carbonitrile-κN1)cobalt(II)]-di-µ-thiocyanato-κ2N:S;κ2S:N] Geometric parameters (Å, º)

**Table:**

Co1—N1^i^	2.068 (2)	C12—H12	0.9300
Co1—N1	2.068 (2)	C12—C13	1.371 (4)
Co1—S2	2.5836 (7)	C13—C14	1.372 (4)
Co1—S2^i^	2.5836 (7)	C13—C16	1.440 (4)
Co1—N11^i^	2.181 (2)	C14—H14	0.9300
Co1—N11	2.181 (2)	C14—C15	1.373 (4)
Co2—S1^ii^	2.6178 (6)	C15—H15	0.9300
Co2—S1^i^	2.6178 (6)	C16—N12	1.132 (4)
Co2—N2^iii^	2.0462 (19)	N21—C21	1.333 (3)
Co2—N2	2.0462 (19)	N21—C25	1.329 (3)
Co2—N21	2.185 (2)	C21—H21	0.9300
Co2—N21^iii^	2.185 (2)	C21—C22	1.376 (4)
N1—C1	1.153 (3)	C22—H22	0.9300
C1—S1	1.636 (2)	C22—C23	1.372 (4)
N2—C2	1.151 (3)	C23—C24	1.374 (4)
C2—S2	1.638 (2)	C23—C26	1.438 (4)
N11—C11	1.333 (3)	C24—H24	0.9300
N11—C15	1.333 (3)	C24—C25	1.372 (4)
C11—H11	0.9300	C25—H25	0.9300
C11—C12	1.380 (4)	C26—N22	1.135 (4)
			
N1^i^—Co1—N1	180.0	C15—N11—Co1	121.45 (17)
N1—Co1—S2	86.30 (6)	C15—N11—C11	117.1 (2)
N1^i^—Co1—S2^i^	86.29 (6)	N11—C11—H11	118.5
N1^i^—Co1—S2	93.70 (6)	N11—C11—C12	123.1 (3)
N1—Co1—S2^i^	93.71 (6)	C12—C11—H11	118.5
N1^i^—Co1—N11^i^	90.63 (8)	C11—C12—H12	120.5
N1^i^—Co1—N11	89.37 (8)	C13—C12—C11	119.0 (3)
N1—Co1—N11	90.63 (8)	C13—C12—H12	120.5
N1—Co1—N11^i^	89.37 (8)	C12—C13—C14	118.5 (3)
S2—Co1—S2^i^	180.00 (2)	C12—C13—C16	121.4 (3)
N11^i^—Co1—S2^i^	87.82 (6)	C14—C13—C16	120.1 (3)
N11^i^—Co1—S2	92.18 (6)	C13—C14—H14	120.5
N11—Co1—S2^i^	92.18 (6)	C13—C14—C15	119.1 (2)
N11—Co1—S2	87.81 (6)	C15—C14—H14	120.5
N11^i^—Co1—N11	180.0	N11—C15—C14	123.3 (2)
S1^ii^—Co2—S1^i^	180.00 (3)	N11—C15—H15	118.4
N2—Co2—S1^i^	92.99 (6)	C14—C15—H15	118.4
N2—Co2—S1^ii^	87.01 (6)	N12—C16—C13	177.9 (4)
N2^iii^—Co2—S1^ii^	92.99 (6)	C21—N21—Co2	121.93 (18)
N2^iii^—Co2—S1^i^	87.01 (6)	C25—N21—Co2	121.07 (17)
N2^iii^—Co2—N2	180.00 (10)	C25—N21—C21	116.9 (2)
N2—Co2—N21^iii^	89.60 (8)	N21—C21—H21	118.5
N2—Co2—N21	90.40 (8)	N21—C21—C22	122.9 (3)
N2^iii^—Co2—N21^iii^	90.40 (8)	C22—C21—H21	118.5
N2^iii^—Co2—N21	89.60 (8)	C21—C22—H22	120.3
N21^iii^—Co2—S1^i^	88.87 (6)	C23—C22—C21	119.3 (3)
N21^iii^—Co2—S1^ii^	91.13 (6)	C23—C22—H22	120.3
N21—Co2—S1^ii^	88.87 (6)	C22—C23—C24	118.3 (3)
N21—Co2—S1^i^	91.13 (6)	C22—C23—C26	121.3 (3)
N21^iii^—Co2—N21	180.0	C24—C23—C26	120.4 (3)
C1—N1—Co1	164.1 (2)	C23—C24—H24	120.7
N1—C1—S1	179.0 (2)	C25—C24—C23	118.6 (3)
C1—S1—Co2^iv^	100.25 (8)	C25—C24—H24	120.7
C2—N2—Co2	166.71 (19)	N21—C25—C24	123.9 (3)
N2—C2—S2	179.5 (2)	N21—C25—H25	118.0
C2—S2—Co1	100.52 (9)	C24—C25—H25	118.0
C11—N11—Co1	121.36 (17)	N22—C26—C23	178.4 (4)
			
Co1—N11—C11—C12	−176.3 (2)	C15—N11—C11—C12	0.6 (5)
Co1—N11—C15—C14	177.0 (2)	C16—C13—C14—C15	179.6 (3)
Co2—N21—C21—C22	−175.4 (2)	N21—C21—C22—C23	0.3 (5)
Co2—N21—C25—C24	175.5 (2)	C21—N21—C25—C24	−0.8 (5)
N11—C11—C12—C13	−1.0 (5)	C21—C22—C23—C24	−1.4 (5)
C11—N11—C15—C14	0.1 (4)	C21—C22—C23—C26	177.7 (3)
C11—C12—C13—C14	0.7 (5)	C22—C23—C24—C25	1.4 (4)
C11—C12—C13—C16	−179.0 (3)	C23—C24—C25—N21	−0.3 (5)
C12—C13—C14—C15	0.0 (4)	C25—N21—C21—C22	0.8 (4)
C13—C14—C15—N11	−0.4 (4)	C26—C23—C24—C25	−177.7 (3)

Symmetry codes: (i) −*x*+2, −*y*+1, −*z*+1; (ii) *x*−1, *y*, *z*−1; (iii) −*x*+1, −*y*+1, −*z*; (iv) *x*+1, *y*, *z*+1.

### catena-Poly[[bis(pyridine-4-carbonitrile-κN1)cobalt(II)]-di-µ-thiocyanato-κ2N:S;κ2S:N] Hydrogen-bond geometry (Å, º)

**Table:**

*D*—H···*A*	*D*—H	H···*A*	*D*···*A*	*D*—H···*A*
C11—H11···N1	0.93	2.63	3.126 (4)	114
C15—H15···N1^i^	0.93	2.59	3.106 (3)	115
C21—H21···N2	0.93	2.61	3.134 (3)	116
C22—H22···N12^v^	0.93	2.67	3.287 (4)	124
C24—H24···S2^vi^	0.93	2.98	3.813 (3)	150
C25—H25···N2^iii^	0.93	2.55	3.065 (4)	115

Symmetry codes: (i) −*x*+2, −*y*+1, −*z*+1; (iii) −*x*+1, −*y*+1, −*z*; (v) *x*, −*y*+1/2, *z*−1/2; (vi) *x*−1, *y*, *z*.

## References

[bb1] Brandenburg, K. (1999). *DIAMOND*. Crystal Impact GbR, Bonn, Germany.

[bb2] Bruno, I. J., Cole, J. C., Edgington, P. R., Kessler, M., Macrae, C. F., McCabe, P., Pearson, J. & Taylor, R. (2002). *Acta Cryst.* B**58**, 389–397. 10.1107/s0108768102003324 12037360

[bb3] Chen, W., Liu, F. & You, X. (2002). *Bull. Chem. Soc. Jpn* **75**, 1559–1560.

[bb4] Clegg, W. & Harrington, R. W. (2016). *CSD Communication* (Refcode UBUBOL). CCDC, Cambridge, England.

[bb5] Groom, C. R., Bruno, I. J., Lightfoot, M. P. & Ward, S. C. (2016). *Acta Cryst*. B**72**, 171–179. 10.1107/S2052520616003954 PMC4822653 27048719

[bb23] Dolomanov, O. V., Bourhis, L. J., Gildea, R. J., Howard, J. A. K. & Puschmann, H. (2009). *J. Appl. Cryst.* **42**, 339–341.

[bb6] Handy, J. V., Ayala, G. & Pike, R. D. (2017). *Inorg. Chim. Acta* **456**, 64–75.

[bb7] Jochim, A., Jess, I. & Näther, C. (2017). *Acta Cryst.* E**73**, 463–466. 10.1107/S205698901700322X PMC5382599 28435698

[bb8] Lescouëzec, R., Toma, L. M., Vaissermann, J., Verdaguer, M., Delgado, F. S., Ruiz-Pérez, C., Lloret, F. & Julve, M. (2005). *Coord. Chem. Rev.* **249**, 2691–2729.

[bb9] Mautner, F. E., Traber, M., Fischer, R. C., Torvisco, A., Reichmann, K., Speed, S., Vicente, R. & Massoud, S. S. (2018). *Polyhedron* **154**, 436–442.

[bb10] Näther, C. (2026). *Acta Cryst.* E**82**, 441–445. 10.1107/S2056989026003373 PMC13148219 42099425

[bb11] Näther, C. & Greve, J. (2003). *J. Solid State Chem.* **176**, 259–265.

[bb12] Neumann, T., Ceglarska, M., Germann, L. S., Rams, M., Dinnebier, R. E., Suckert, S., Jess, I. & Näther, C. (2018). *Inorg. Chem.* **57**, 3305–3314. 10.1021/acs.inorgchem.8b00092 29505252

[bb13] Rams, M., Jochim, A., Böhme, M., Lohmiller, T., Ceglarska, M., Rams, M. M., Schnegg, A., Plass, W. & Näther, C. (2020). *Chem. Eur. J.* **26**, 2837–2851. 10.1002/chem.201903924 PMC7078958 31702081

[bb14] Sheldrick, G. M. (2008). *Acta Cryst.* A**64**, 112–122. 10.1107/S0108767307043930 18156677

[bb15] Sheldrick, G. M. (2015*a*). *Acta Cryst.* A**71**, 3–8.

[bb16] Sheldrick, G. M. (2015*b*). *Acta Cryst.* C**71**, 3–8.

[bb17] Stoe (2008). *X-AREA*, *X-RED32* and *X-SHAPE*. Stoe & Cie, Darmstadt, Germany.

[bb18] Wellm, C., Neumann, T., Gallo, G., Dziubyna, A. M., Rams, M., Dinnebier, R. E. & Näther, C. (2020). *Cryst. Growth Des.* **20**, 3374–3385.

[bb19] Westrip, S. P. (2010). *J. Appl. Cryst.* **43**, 920–925.

[bb20] Wöhlert, S., Fic, T., Tomkowicz, Z., Ebbinghaus, S. G., Rams, M., Haase, W. & Näther, C. (2013). *Inorg. Chem.* **52**, 12947–12957. 10.1021/ic4012235 24171470

[bb21] Wöhlert, S., Ruschewitz, U. & Näther, C. (2012). *Cryst. Growth Des.* **12**, 2715–2718.

[bb22] Wriedt, M. & Näther, C. (2010). *Chem. Commun.* **46**, 4707–4709. 10.1039/c0cc00064g 20583337

